# Weekly self-measurement of FEV1 and PEF and its impact on ACQ (asthma control questionnaire)-scores: 12-week observational study with 76 patients

**DOI:** 10.1038/s41533-017-0064-4

**Published:** 2017-12-08

**Authors:** Christoph Ulrich Werner, Klaus Linde, Julia Schäffner, Constanze Storr, Antonius Schneider

**Affiliations:** Technical University of Munich, TUM School of Medicine, Institute of General Practice, Munich, Germany

## Abstract

The “Asthma Control Questionnaire” (ACQ) is a very common questionnaire for assessing asthma control. This study compares different ACQ versions in a self-monitoring program over a 12-week period combining them with patients' self-measurements of peak expiratory flow (PEF) and forced expiratory volume in one second (FEV1). The objective was to test the feasibility of FEV1-self-measurements and to compare ACQ versions regarding possible additional information given by lung function. In this prospective multicenter observational study 100 adult asthma patients, recruited at six family practices and two pulmologists' private practices in Germany, completed the ACQ weekly, performing self-measurements of PEF and FEV1. Seventy-six patients were included into final analysis with only 3% missing values. Scores for all ACQ versions improved significantly (all *P*-values < 0.05) with reductions of 32% for ACQ5, 31% for ACQ6, 22% for ACQ7-FEV1, and 21% for ACQ7-PEF with high Pearson’s correlation coefficients of all scores (*r* between 0.96 and 0.99). ACQ7-FEV1 scores were significantly higher than others. Separated courses of lung function parameters showed nearly no change, but ACQ5 and ACQ6 as scores for symptoms and reliever medication improved constantly. ACQ5 and ACQ6 revealed higher percentages of patients classified as “controlled” than ACQ7-scores. In conclusion, with only a few missing data points, our results suggest feasibility of FEV1-self-measurements. Courses of symptom-related and lung function-related ACQ items differ clearly. Our results support the GINA recommendations to consider symptoms and lung function separately. FEV1-self-measurements for research purposes may be included with the ACQ, but in clinical practice seem to measure a different domain to symptomatic asthma control.

## Asthma: self-measurement of lung function is feasible

Lung function tests taken by patients at home is doable, but yields different information than self-reports of disease control. Christoph Werner and colleagues from the Technical University of Munich, Germany, tested the feasibility of collecting lung function data from patients who measured forced expiratory volume in one second by themselves. These same patients also completed the Asthma Control Questionnaire (ACQ), which describes symptom severity and medication use. Looking at data from 76 participants, the researchers found that people did well measuring forced expiratory volume in one second, but they could not say whether this test was superior to another metric of lung function that considers peak expiratory flow. The results also showed that symptom scores and lung function assays seem to capture different aspects of the disease, meaning they’re worth considering separately rather than lumping together in a single test.

## Introduction

In long-term management of patients suffering from bronchial asthma the key target is the control of the disease.^1–5^ This so-called “asthma control” is understood as the extent to which clinical manifestations of the disease can be or have been reduced or even eradicated by treatment.^6^ Assessing asthma control has two domains:^7^ These are on the one hand the clinical control (consisting of current clinical symptoms and the capability of patients continuing daily activities and reaching a maximum quality of life) and on the other hand, risk factors for adverse events like exacerbations.^1^

Therefore, in clinical routine assessing asthma control means to measure the patients' clinical symptoms and lung function parameters. To assess symptoms, there are several questionnaires in use, which ask about the frequency of typical asthma symptoms and use of reliever medications.^8–10^ Juniper et al. developed one of these questionnaires, the “Asthma Control Questionnaire” (ACQ), in 1999. Since then it is in use in a large number of countries being translated in 115 languages so far.^8,11^ This questionnaire is available in several versions. The original version includes not only questions about symptoms and reliever medication, but also an item on lung function. The authors of the ACQ recommend preferring measurements of FEV1 to measurements of peak expiratory flow (PEF) in usage with the ACQ, as the questionnaire has been validated with FEV1, but for practical reasons in daily disease management, they also accept PEF.^11^ Due to technical reasons, in the past it has not been possible for patients to perform self-measurements of FEV1 themselves at home. Still only a few but expensive portable devices are available. For all ACQ versions – whether including an item on lung function or not – an overall summary score is calculated. Previous studies, one of them done by Juniper et al. themselves, compared different ACQ versions and found extremely high correlation coefficients suggesting that adding or leaving out lung function makes little difference.^12–16^

In the 2015 update of its strategy for asthma management and prevention the “Global Initiative for Asthma” (GINA) advises to consider symptoms and lung function parameters separately, with lung function being no longer included among symptom measures numerically, but being mainly used for further risk assessment.^7^ In its 2017 update, GINA recommends long-term lung function-monitoring (PEF) for severe asthma cases and patients with poor perception of air flow limitation only.^17^

In the study described below, we aimed to investigate, if weekly FEV1-measurements performed by patients at home over a 12 weeks period are feasible. Furthermore, we compared different versions of the ACQ to investigate whether inclusion of lung function provides additional relevant information, and whether it makes a difference if FEV1 is used instead of PEF.

## Results

### Exclusions from/inclusions to analysis

Of the 100 patients, who gave consent and were provided with asthma questionnaires and measurement devices at the beginning, 80 sets of records were returned. Three sets of records had been handed out to under-aged persons by mistake. Two patients quit the study prematurely because they spontaneously were free of symptoms. Six participants did not return their questionnaires and could not be contacted. Nine sets of records got lost by practices or patients. Furthermore, four more patients were excluded from the study: one patient had been given questionnaires because of his medical history only, but indeed had not been diagnosed securely, by neither a pulmologist nor a GP. For three patients no baseline values for ACQ7-FEV1 could be calculated. Finally, 76 patients were included into analysis. Overall, questionnaires were filled in very carefully by patients with only 3% missings of a total of 9880 item values. However, in 27 patients one or more ACQ items were missing for at least 1 week. 49 patients (64%) had filled in their sets of records without any missing data. The online supplementary tables provide a detailed overview on data available for the individual ACQ items and summary scores.

### Description of study participants

Characteristics of study participants are shown in Table 1. Average age of patients was 45.6 years with 80% being under 60 years of age. 57 (75%) were female and 19 (25%) male. Mean BMI of study participants was 26.0 kg/m^2^. Fourteen persons (18%) were smokers. Eighteen patients (24%) had been diagnosed with asthma by a GP and 58 patients (76%) by a pulmologist. Fifty-two (68%) had undergone an asthma training program prior to the study with 92% of those participants being current non-smokers. 36 patients (47%) were recruited at general practitioners' practices, 40 (53%) at pulmologists' practices. The mean ACQ6 value at baseline was 1.3 with 34 (45%) of participants presenting with controlled asthma, mean FEV1 %-predicted in week 1 was 80.5%. In all, 92% were using a controller medication with 83% taking an inhalative corticosteroid, some in combination with other controllers. 59% took a reliever medication, one patient did not take any medication. Qualitative review of medication at baseline revealed an exceptional variety of treatment strategies. Some strategies seemed inconsistent with seven participants' treatment being incompatible with any guidelines.

**Table 1:** Characteristics of participants (*n* = 76. values are absolute frequencies or means)

Overall	76 (100%)
Women	57 (75%)
Diagnosed by pulmologist	58 (76%)
Diagnosed by GP	18 (24%)
Recruited by specialist	36 (47%)
Recruited by GP	40 (53%)
Smokers	14 (18%)
Mean age (overall)	45.6 years (SD = 15.4)
Mean BMI (overall)	26.0 mg/m^2^ (SD = 5.0)
Previous asthma training	52 (68%)
*ACQ 6 week 1*	
Mean	1.3 (SD 1.0)
≤0.75	34 (45%)
0.76–1.49	19 (25%)
≥1.5	23 (30%)
FEV1 mean week 1	2.4 (SD 0.7)
FEV1 %-predicted mean week 1	80.5% (SD 19.1%)
PEF mean week 1 (mornings/evenings)	404 (SD 96) /390 (SD 98)
PEF %-predicted mean week 1	90.9% (SD 21.9%)
*Asthma medication*	
ICS	63 (83%)
LABA	45 (59%)
RABA	52 (68%)
Others	11 (15%)
Controller	70 (92%)
Reliever	45 (59%)
Controller + reliever	38 (50%)
None	1 (1.3%)

*GP* general practitioner, *BMI* body mass index, *SD* standard deviation, ACQ 6 = Asthma Control Questionnaire version with 6 items, FEV1 = forced expiratory volume in 1 second, *PEF* peak expiratory flow, *ICS* inhalative corticosteroid, *LABA* long-acting beta-2 agonist, *RABA* rapid-acting beta-2 agonist

### Clinical measurements

**Fig. 1:**
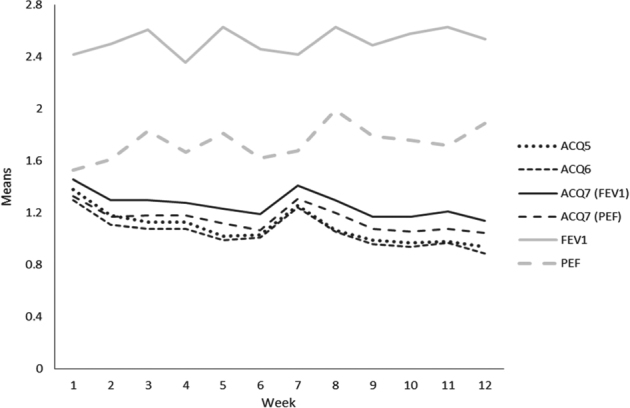
Means of summary scores of the four versions of the ACQ (black lines) and of the single ACQ-items for FEV1-%predicted and PEF %-predicted (grey lines) from week 1 to week 12, ACQ-scores for single items and summary scores range from 0 to 6. (Analyses are based on available data per week (number of available observations between 69 and 75). Standard errors for the single measurement points range between 0.1 and 0.2)

Over the 12-week period summary scores for all four versions of the ACQ improved significantly, both in complete cases and when replacing missing values (all *p*-values < 0.05; Fig. 1; see supplementary Table 1 for detailed descriptive statistics).

Compared with baseline at week 1 mean score at week 12 was 32% lower for the ACQ5, 31% for the ACQ6, 22% for the ACQ7 if using FEV1 % predicted, and 21% for ACQ7 if using PEF % predicted. Throughout the study summary scores for ACQ7-FEV1 were significantly (*p* < 0.05 for every single week) higher than for all other ACQ scores. ACQ–PEF summary scores tended to be higher than ACQ5 and ACQ6 scores but differences were not always significant. Yet, Pearson’s correlation coefficients of the four ACQ summary scores were very high (*r* between 0.96 and 0.99; see Table 2, columns two to four, showing week 2 data as an example). Crohnbach's alpha was highest for the ACQ5 (0.89 in week 2), and slightly decreased by adding the medication (0.87) and lung function (0.85 for both FEV1 and PEF).

**Table 2:** Pearson correlation coefficients (*r*) comparing different versions of the ACQ, the ACQ item for puffs and lung function measurements at week 2 (number of valid observations between 73 and 76)

*R* for	ACQ6	ACQ7 (FEV1%P)	ACQ7 (PEF%P)	ACQ item puffs	FEV1 raw value	FEV1 %P	FEV1 %P coded ACQ	PEF raw value	PEF %P	PEF %P coded ACQ
ACQ5	0.99	0.96	0.96	0.48	−0.52	−0.46	0.49	−0.42	−0.43	0.45
ACQ6		0.97	0.97	0.57	−0.51	−0.43	046	−0.44	−0.41	0.44
ACQ7 (FEV1 %P)			0.97	0.58	−0.63	−0.81	0.67	−0.51	−0.54	0.54
ACQ (PEF %P)				0.56	−0.54	−0.51	0.56	−0.58	−0.61	0.64
ACQ item puffs					−0.27*	−0.31*	0.35*	−0.22#	−0.28*	0.27*
FEV1 % raw value						0.67	−0.71	0.69	0.45	−0.41
FEV %P							−0.91	0.42	0.60	−0.55
FEV1 %P coded ACQ								−0.50	−0.69	0.64
PEF raw value									0.81	−0.75
PEF %P										−0.93

%P = % predicted, correlations are statistically significant at the *p* < 0.001 except * which are significant at *p* < 0.05 but ≥ 0.001 and # which are not significant (*p* ≥ 0.05)

**Fig. 2:**
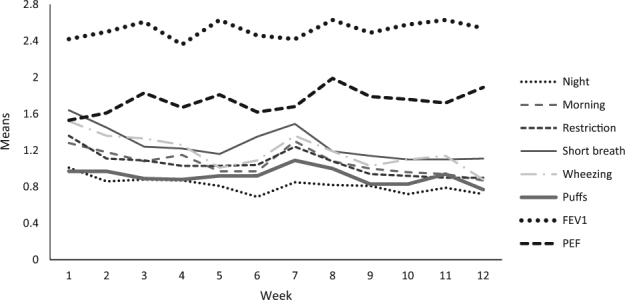
Means of the single ACQ items from week 1 to week 12, scores for single ACQ-items range from 0 to 6. Analyses are based on available data per week (number of available observations between 71 and 76). Standard errors for the single measurement points range between 0.1 and 0.2

Diverse courses emerged from analysis over the 12-week period of our study if considering ACQ scores for items of single symptoms, puffs of relievers taken and lung function parameters separately (Fig. 2; see supplementary Tables 2 and 3 for detailed descriptive statistics). Means of raw values of FEV1 and PEF %-predicted remained almost stable over time, but results for FEV1 were about 10% worse than for PEF (data not shown). Transformation of raw values into ACQ item scores resulted in considerably higher values for the item ACQ-FEV1 than for the item ACQ–PEF and, accordingly, higher ACQ7-FEV1 than ACQ7-PEF scores (see Figs. 1 & 2). In contrast to lung function parameters, mean values for single symptoms and bronchodilator use decreased over the time (Fig. 2).

This led to an increasing difference in the courses of ACQ scores and isolated courses of FEV1 and PEF (biggest difference to ACQ seen in FEV1) (Fig. 1). Pearson correlation coefficients between the symptoms summarized in ACQ5 and puffs or lung function values were moderate. Table 2 (columns 5–10) provides week 2 data as an example. Pearson’s *r* was 0.48 for the correlation between ACQ5 and puffs, and varied between 0.42 and 0.52 for lung function (variable algebraic signs due to scaling). Findings for the other ACQ versions were similar. Correlations between puffs and lung function were low to moderate (*r* between 0.22 and 0.35 for week 2). Results were similar if evening measurements were analyzed instead of morning measurements.

**Fig. 3:**
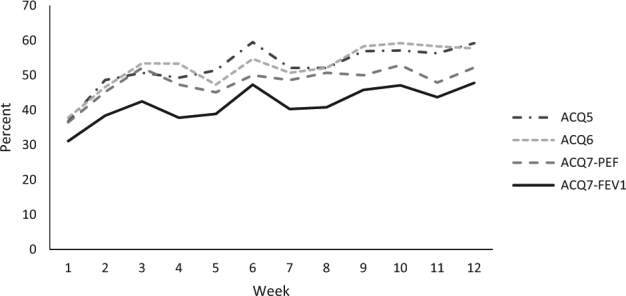
Proportion of participants with asthma classified as controlled (≤0.75) according to different ACQ versions, indicated in percent from week 1 to week 12. Analyses are based on available data per week (number of available observations between 69 and 75)

Classifying the study population as “controlled”, “partly controlled” and “uncontrolled” by using ACQ7-FEV1 over the 12-week period there was an increasing size of the “controlled” group and a decrease of the “partly controlled”, whereas the “uncontrolled” nearly stayed the same. Again, while showing similar courses for all four ACQ versions, ACQ5 and ACQ6 resulted in higher percentages of “controlled” patients compared with the ACQ scores with lung function parameters, with the lowest percentage to be seen for ACQ7-FEV1 (Fig. 3). We did not identify single patients and their eventual switches between groups because of possible exacerbations, but during the 12-week period of our study no patient severely exacerbated with the need for oral glucocorticoids or even hospitalization.

## Discussion

### Main findings

In this study the majority of participants managed well to carry out FEV1-self-measurements together with the ACQ weekly at home over a period of 12 weeks. Summary scores of all four versions of the ACQ improved over time, showing very high correlation. Similar development was seen if classifying patients in control groups, but ACQ5 and ACQ6 classifying higher percentages as “controlled”. However, ACQ7-FEV1 summary scores were significantly higher than those of all other ACQ versions throughout the study. FEV1 values were consistently worse than PEF values. Investigating the courses of single ACQ items showed that both FEV1 and PEF remained stable over time, meaning that the reduction in summary scores was mainly driven by improving ratings of asthma symptoms.

### Strengths and limitations

This study was conducted to investigate the course of asthma control in a naturalistic sample of patients doing regular FEV1-self-measurements at home. So far, repeated self-measurements by patients were carried out for PEF only. The study protocol allows a detailed analysis of ACQ-changes over time. The findings are internally highly consistent. Our findings on feasibility have to be interpreted on special regard to the fact that 24 patients giving consent to the study could not be included into analysis for various reasons. Especially the number of 15 not returned or lost questionnaires could have been caused by the repeated measurements possibly being burdensome to patients. Also, the incentives (20€ and keeping the device) are likely to have increased adherence. Finally, we assessed feasibility only by counting missing. It could also be discussed, if improvement in asthma of some of the participants was derived from changes in their medication made at baseline visit. But as every patient diagnosed with asthma earlier on was offered to participate in the study, not only if consulting the doctor for problems with their asthma but for whatever reason, we think our study population represents a realistic sample of typical asthma patients with typical courses of their disease. Also, because of the broad selection criteria it is likely that patients represent typical asthma outpatients in the German health care system, although our sample of patients is not very large. Another argument against a medication derived change during the study is the persisting difference of lung function and symptom scores with lung function getting even slightly worse over a period of 12 weeks, which clearly stands against the improving symptom score only being caused by an effective change in medication. Within our study, it was not possible to investigate the validity of lung function self-measurements, e.g. by comparing these to regular spirometry in physicians' practices, but another study showed no significant difference between FEV1 at home and FEV1 using official spirometry at a physicians' offices (even though all other ACQ7-items did differ significantly).^18^

### Interpretation of findings in relation to previously published work

The very high correlation in scores of all ACQ versions meets the results of previous studies, which also presented usability of ACQ versions with and without lung function without loss of validity or change in interpretation.^12–16^ At the same time scores for symptoms and need for reliever medication improved significantly in contrast to both absolute values and the course over time of ACQ items for lung function, in particular for FEV1, which showed constant courses with even non-significant worsening (FEV1 showed 10% worse results than PEF). A recent study reporting a factorial analysis of ACQ6 and ACQ7 (with FEV1) found that the FEV1 item showed no relation to the latent factor derived from all other items and concluded that the factor structure of the ACQ7 remains unclear.^19^ It is likely that the very high correlations between different ACQ versions are mainly owing to the fact, that the majority of items (symptoms) is the same in all versions, with FEV1/PEF being only one of seven items. Lung function items do correlate with the other items but only moderately or weakly. They only have a little impact on ACQ7-scores, which rather mainly are influenced by patients' subjective concerns about their symptoms and by their reactions using their emergency devices according to emergency/self-management plans. The question is therefore, why put in this effort doing lung function measurements at all. So to us, it seems likely that symptomatic and lung function items could measure different dimensions of asthma control or disease status: on the one hand the subjective perceived present clinical control presented by symptoms and need of reliever medication, on the other hand the more objective unadulterated aspect of control, measured by lung function which may be an indicator of future risk regarding patients' prognosis for the disease.

### Implications for future research, policy and practice

If this would be true, it would have potential consequences for the use of the ACQ. The very strong correlation between all four ACQ versions is raising the question, why lung function measurements should be done at all, if being integrated in summary scores according to current rules:

- Previous reviews showed equal benefits for patients using self-management programs with PEF-measurements or with symptom scores.^20,21^ It could be considered to weight lung function items as such of overriding importance, so they would have more impact on ACQ7.
- Currently, the same coding rules are used for transforming raw values of FEV1 %-predicted and PEF %-predicted into an ACQ item. Maybe this should be changed, because in repeated observations FEV1 %-predicted on average was about 10% worse than PEF %-predicted if coded in the same way.^20^ But even as there is a lack of studies for FEV1, this is very questionable for both parameters if following the results of another review, which showed improving health outcomes for patients using action plans based on personal best PEF, in contrast to patients using plans based on PEF %-predicted.^22^
- A third alternative would be to present the ACQ6 summary score and lung function separately: the difference in courses of symptom scores and lung function items could be caused by on the one hand patients' individual and subjective perception of symptoms and deterioration. It is likely that improving scores of ACQ5 and ACQ6 express the effect of patients getting used to the procedure, e.g., as a learning effect in responding questions in a diary repeatedly. This can result in understanding the questions better and subsequently assigning better scores. Improving scores for symptoms and reliever medication can also be caused by patients getting used to stable asthma control and so being confirmed in their efforts positively. For lung function, this effect is not possible because you have to write down a value, which is not subjective.
  On the other hand, the stable courses of lung function parameters could be interpreted as a sign of these items being a helpful because objective and so unadulterated parameters in assessing asthma control. A previous study showed a discordance in patients' perception of asthma control and their actual asthma control comparing their personal impressions and perceptions with the results of another questionnaire to measure asthma control, the “Asthma Control Test”, with a high percentage of patients feeling controlled despite their test results showing an uncontrolled asthma.^23^ Implying this into interpretation of our study's results gives additional support to consider symptom scores and lung function separately.

Following this argumentation, we think symptoms and lung function both should be measured in clinical studies using the ACQ, for example, when investigating whether an intervention or treatment modifies only one or both of these aspects.

It could be questioned if in these cases there should be PEF- or FEV1-measurements or both. Because FEV1%-predicted and PEF %-predicted have been shown to differ systematically,^24^ it seems to be appropriate measuring both in clinical studies using the ACQ and rectifies additional costs by an expectable gain of knowledge. In self-management plans for patients ACQ so far has been used with PEF, although it was developed with FEV1, just because of regular FEV1-measurements being too expensive. More than this, there seems to be no additional benefit for patients from self-management plans with PEF-self-measurements than those involving symptoms only.^20,21^ Unless randomized trials could prove that interventions involving self-measurement of FEV1 lead to better patient-relevant outcomes, there is little argument for advocating their use.

In conclusion, we cannot give any recommendation to prefer FEV1 to PEF in use with the ACQ in clinical studies, but we think in connection with the ACQ lung function is more suitable for use in studies, where you can use a device measuring both, than for daily routine.

## Conclusions

The results of this study show the feasibility of regular FEV1-self-measurements performed by patients themselves at home monitoring their asthma control. However, following our data no recommendation can be given for preferring FEV1- to PEF-measurements. More than this, our results lend further support to the GINA recommendation to look at symptom scores and lung function separately. Further studies should investigate whether the ACQ7 including a lung function item should be considered a two-dimensional instrument. Self-monitoring of FEV1 is feasible, and for research purposes may be included with the ACQ questions, but in clinical practice measures a different domain to symptomatic asthma control.

## Methods

### Ethics

The study was approved by the Ethics Committee of the Medical Faculty of the Technical University Munich. It was run according to all relevant guidelines and procedures, especially in accordance to the professional code of medical doctors, the Declaration of Helsinki in its 2008 version and to the German Federal Data Protection Act. All patients gave written consent to their participation.

### Patient recruitment and inclusion criteria

This was a 12-week prospective multicenter observational study conducted by the Institute of General Practice of the Technical University Munich, Germany in 2011. Patients were recruited at six family practices and two pulmologists' private practices. All consecutive patients with a diagnosis of asthma consulting study practices for whatever reason who met inclusion criteria were informed about the study and offered to participate. Inclusion criteria were age ≥ 18 years, asthma diagnosis confirmed by spirometry in family practice or by a pulmologists via spirometry or bronchial provocation, signed declaration of informed consent and sufficient knowledge of German language. Patients were offered an incentive of €20 each for completing their asthma questionnaires. In addition, they could keep the used digital FEV1- and Peak-Flow-Meter (worth €70) after the study was finished.

### Asthma control questionnaire

For assessment of asthma control the German version of the ACQ was used.^25^ This questionnaire consists of five questions regarding the most frequent asthma symptoms in the previous week (nocturnal awakening, severity of symptoms in the morning, limitation in daily activities, frequency of dyspnea and frequency of wheezing), one question regarding puffs of reliever medication and one question regarding lung function parameters before use of a bronchodilator.^8^ For every question, a score from zero to six is set up. For lung function parameters, measurements of FEV1 or PEF are transferred into percentage of nominal value and then into the same score from zero (observed values > 95% of predicted value) to six (<50%). By adding all scores for single items and dividing the result by the number of items, summary scores are calculated to define the degree of asthma control. In our study, scores were calculated for four different versions of the ACQ: the ACQ5, consisting of five questions about symptoms only, the ACQ6, consisting of ACQ5 and one question about use of reliever medication, ACQ7-PEF, consisting of ACQ6 and PEF-self-measurements and ACQ7-FEV1, same as ACQ7-PEF, but conducted with FEV1-self-measurements instead. Cutoff points for asthma control are ≤ 0.75 for controlled asthma, 0.76–1.49 for partly controlled asthma and ≥ 1.5 for uncontrolled asthma and can be used the same for all four versions.^26^

### Study process

Both, FEV1- and PEF-measurements were done by the patients themselves with the digital peak-flow-meter Vitalograph asma-1-monitor, which is a high quality and very reliable device saving up to 600 measurements. In the first week, patients measured FEV1 and PEF twice a day, mornings and evenings, by taking the best results out of three measurements at each time. This procedure was done to get reliable baseline values and to get patients used to the electronic device and possible differences to the readings by their usual mechanical devices. At the end of week one they answered the ACQ and made optional comments.

From week 2–12, patients did their measurements of FEV1 and PEF in the mornings and evenings once a week only, again taking the best of three results, completing the ACQ again once a week. The ACQ7-scores for week one were set up using the means of FEV1- und PEF-measurements in the morning. In weeks 2–12, the weekly gathered measurements in the morning were used. Besides, patients reported their medication use per week and important events like hospitalization. In addition, participants received an individual self-management-plan for adjusting their medication etc. in case of a worsening of their asthma including an individual emergency action plan. There was no further planned intervention such as control appointments or phone calls, except in case of exacerbations, when patients consulted the doctors. Severe exacerbations were defined by the need for oral glucocorticoids or even hospitalization.^27,28^

### Data analysis

Data were entered into and processed with SPSS using all available data. Descriptive analysis was done on data using means (standard deviations) or absolute frequencies (percentages). Changes in ACQ scores over the 12-week period were analyzed using repeated measures analysis of variance. As about a quarter of the patients at some point had a missing value, we used multiple imputation (five runs) to investigate the influence of missing data. Pearson coefficients were used to quantify correlations. Consistency of the four ACQ scores was tested with Crohnbach’s α. The paired *t*-test was used to explore whether the different ACQ summary scores differed between each other for single weeks. A formal sample size calculation was not performed. Methods were performed in accordance with relevant regulations and guidelines.

### Data availability

Prior to the performing of the study, we guaranteed to our patients that their data would not be transmitted outside, even not anonymously. Therefore, our medical ethics committee considers data sharing in this case as problematic.

## Electronic supplementary material

Supplementary tables
