# Supplementary material for: Weekly self-measurement of FEV1 and PEF and its impact on ACQ (asthma control questionnaire)-scores: 12-week observational study with 76 patients
Source: NPJ Prim Care Respir Med. 2017 Dec 8;27:64. doi: 10.1038/s41533-017-0064-4 (PMC5722863; doi:10.1038/s41533-017-0064-4)
Supplement: Supplementary file 1 — Supplementary tables [file 41533_2017_64_MOESM1_ESM.docx]

Supplementary Table 1: Descriptive statistics and 95% CIs (LL = lower limit, UL = upper limit) for the four ACQ versions

|  | Week 1 | Week 2 | Week 3 | Week 4 | Week 5 | Week 6 | Week 7 | Week 8 | Week 9 | Week 10 | Week 11 | Week 12 |
| --- | --- | --- | --- | --- | --- | --- | --- | --- | --- | --- | --- | --- |
| **ACQ5** |  |  |  |  |  |  |  |  |  |  |  |  |
| N | 75 | 74 | 73 | 75 | 74 | 74 | 73 | 73 | 72 | 70 | 71 | 71 |
| Mean | 1.38 | 1.18 | 1.13 | 1.13 | 1.02 | 1.03 | 1.26 | 1.07 | 0.98 | 0.97 | 0.98 | 0.94 |
| Std. Error of Mean | 0.12 | 0.13 | 0.14 | 0.13 | 0.13 | 0.14 | 0.15 | 0.14 | 0.13 | 0.14 | 0.12 | 0.12 |
| Std. Deviation | 1.08 | 1.09 | 1.23 | 1.12 | 1.08 | 1.21 | 1.25 | 1.17 | 1.08 | 1.15 | 1.05 | 1.02 |
| 95%CI LL | 1.14 | 0.94 | 0.85 | 0.88 | 0.77 | 0.76 | 0.98 | 0.80 | 0.74 | 0.70 | 0.74 | 0.70 |
| 95%CI UL | 1.62 | 1.43 | 1.42 | 1.39 | 1.26 | 1.31 | 1.55 | 1.34 | 1.23 | 1.23 | 1.22 | 1.18 |
| **ACQ6** |  |  |  |  |  |  |  |  |  |  |  |  |
| N | 74 | 73 | 73 | 75 | 74 | 75 | 73 | 73 | 72 | 71 | 72 | 71 |
| Mean | 1.30 | 1.10 | 1.08 | 1.08 | 0.99 | 1.01 | 1.24 | 1.06 | 0.96 | 0.94 | 0.97 | 0.89 |
| Std. Error of Mean | 0.11 | 0.11 | 0.13 | 0.12 | 0.12 | 0.13 | 0.14 | 0.13 | 0.12 | 0.13 | 0.12 | 0.11 |
| Std. Deviation | 0.98 | 0.94 | 1.13 | 1.05 | 1.00 | 1.17 | 1.21 | 1.10 | 1.02 | 1.09 | 1.00 | 0.95 |
| 95%CI LL | 1.07 | 0.89 | 0.82 | 0.84 | 0.76 | 0.75 | 0.96 | 0.81 | 0.72 | 0.68 | 0.74 | 0.67 |
| 95%CI UL | 1.52 | 1.32 | 1.34 | 1.32 | 1.22 | 1.27 | 1.51 | 1.31 | 1.19 | 1.19 | 1.20 | 1.11 |
| **ACQ7 (FEV1)** |  |  |  |  |  |  |  |  |  |  |  |  |
| N | 74 | 73 | 73 | 74 | 72 | 74 | 72 | 71 | 72 | 70 | 71 | 69 |
| Mean | 1.46 | 1.30 | 1.30 | 1.28 | 1.23 | 1.19 | 1.41 | 1.30 | 1.17 | 1.18 | 1.21 | 1.14 |
| Std. Error of Mean | 0.12 | 0.11 | 0.13 | 0.12 | 0.12 | 0.13 | 0.14 | 0.13 | 0.12 | 0.13 | 0.12 | 0.12 |
| Std. Deviation | 0.99 | 0.96 | 1.12 | 1.03 | 1.02 | 1.11 | 1.15 | 1.06 | 1.02 | 1.06 | 1.02 | 0.97 |
| 95%CI LL | 1.23 | 1.08 | 1.04 | 1.05 | 1.00 | 0.94 | 1.15 | 1.05 | 0.94 | 0.93 | 0.97 | 0.92 |
| 95%CI UL | 1.69 | 1.52 | 1.55 | 1.51 | 1.47 | 1.45 | 1.68 | 1.54 | 1.41 | 1.43 | 1.45 | 1.37 |
| **ACQ7 (PEF)** |  |  |  |  |  |  |  |  |  |  |  |  |
| N | 74 | 73 | 73 | 74 | 71 | 74 | 72 | 71 | 72 | 70 | 71 | 69 |
| Mean | 1.33 | 1.17 | 1.18 | 1.18 | 1.12 | 1.07 | 1.31 | 1.20 | 1.08 | 1.06 | 1.08 | 1.05 |
| Std. Error of Mean | 0.12 | 0.11 | 0.13 | 0.12 | 0.12 | 0.13 | 0.14 | 0.13 | 0.12 | 0.13 | 0.12 | 0.12 |
| Std. Deviation | 1.00 | 0.95 | 1.13 | 1.03 | 1.03 | 1.12 | 1.18 | 1.10 | 1.01 | 1.07 | 1.04 | 1.00 |
| 95%CI LL | 1.11 | 0.95 | 0.92 | 0.95 | 0.89 | 0.82 | 1.03 | 0.95 | 0.84 | 0.81 | 0.84 | 0.81 |
| 95%CI UL | 1.56 | 1.39 | 1.44 | 1.41 | 1.36 | 1.33 | 1.58 | 1.46 | 1.31 | 1.31 | 1.33 | 1.28 |

Supplementary Table 2: Descriptive statistics and 95% CIs (LL = lower limit, UL = upper limit) for individual ACQ items (part 1)

|  | Week 1 | Week 2 | Week 3 | Week 4 | Week 5 | Week 6 | Week 7 | Week 8 | Week 9 | Week 10 | Week 11 | Week 12 |
| --- | --- | --- | --- | --- | --- | --- | --- | --- | --- | --- | --- | --- |
| **FEV1** |  |  |  |  |  |  |  |  |  |  |  |  |
| N | 76 | 76 | 76 | 75 | 73 | 74 | 73 | 73 | 73 | 72 | 72 | 70 |
| Mean | 2.42 | 2.50 | 2.61 | 2.36 | 2.63 | 2.46 | 2.42 | 2.63 | 2.49 | 2.58 | 2.63 | 2.54 |
| Std. Error of Mean | 0.22 | 0.22 | 0.22 | 0.22 | 0.22 | 0.22 | 0.22 | 0.22 | 0.23 | 0.22 | 0.23 | 0.24 |
| Std. Deviation | 1.89 | 1.91 | 1.96 | 1.88 | 1.84 | 1.87 | 1.89 | 1.88 | 2.01 | 1.84 | 1.97 | 2.02 |
| 95%CI LL | 2.00 | 2.07 | 2.16 | 1.93 | 2.21 | 2.03 | 1.99 | 2.20 | 2.03 | 2.16 | 2.17 | 2.07 |
| 95%CI UL | 2.84 | 2.93 | 3.05 | 2.79 | 3.05 | 2.89 | 2.86 | 3.06 | 2.95 | 3.01 | 3.08 | 3.02 |
| **PEF** |  |  |  |  |  |  |  |  |  |  |  |  |
| N | 76 | 76 | 76 | 75 | 72 | 74 | 73 | 73 | 73 | 72 | 72 | 70 |
| Mean | 1.53 | 1.61 | 1.83 | 1.67 | 1.81 | 1.62 | 1.68 | 1.99 | 1.79 | 1.76 | 1.72 | 1.89 |
| Std. Error of Mean | 0.22 | 0.21 | 0.20 | 0.21 | 0.23 | 0.22 | 0.24 | 0.24 | 0.23 | 0.23 | 0.24 | 0.24 |
| Std. Deviation | 1.89 | 1.80 | 1.78 | 1.80 | 1.92 | 1.94 | 2.05 | 2.02 | 1.99 | 1.96 | 2.01 | 2.02 |
| 95%CI LL | 1.10 | 1.20 | 1.43 | 1.26 | 1.36 | 1.18 | 1.22 | 1.52 | 1.34 | 1.31 | 1.26 | 1.41 |
| 95%CI UL | 1.95 | 2.01 | 2.23 | 2.07 | 2.25 | 2.06 | 2.15 | 2.45 | 2.25 | 2.22 | 2.19 | 2.36 |
| **Puffs** |  |  |  |  |  |  |  |  |  |  |  |  |
| N | 74 | 74 | 76 | 76 | 75 | 75 | 74 | 73 | 72 | 71 | 72 | 71 |
| Mean | 0.97 | 0.97 | 0.89 | 0.88 | 0.92 | 0.92 | 1.09 | 1.00 | 0.83 | 0.83 | 0.94 | 0.77 |
| Std. Error of Mean | 0.11 | 0.10 | 0.12 | 0.12 | 0.13 | 0.14 | 0.15 | 0.13 | 0.12 | 0.13 | 0.13 | 0.11 |
| Std. Deviation | 0.94 | 0.89 | 1.05 | 1.03 | 1.09 | 1.24 | 1.27 | 1.09 | 0.99 | 1.11 | 1.10 | 0.96 |
| 95%CI LL | 0.76 | 0.77 | 0.66 | 0.65 | 0.67 | 0.64 | 0.80 | 0.75 | 0.60 | 0.57 | 0.69 | 0.55 |
| 95%CI UL | 1.19 | 1.18 | 1.13 | 1.11 | 1.17 | 1.20 | 1.38 | 1.25 | 1.06 | 1.09 | 1.20 | 1.00 |
| **Wheezing** |  |  |  |  |  |  |  |  |  |  |  |  |
| N | 75 | 76 | 76 | 76 | 75 | 75 | 74 | 73 | 72 | 71 | 72 | 71 |
| Mean | 1.52 | 1.36 | 1.33 | 1.26 | 1.01 | 1.09 | 1.36 | 1.18 | 1.03 | 1.10 | 1.14 | 0.99 |
| Std. Error of Mean | 0.19 | 0.17 | 0.19 | 0.15 | 0.14 | 0.16 | 0.17 | 0.16 | 0.16 | 0.17 | 0.17 | 0.14 |
| Std. Deviation | 1.62 | 1.51 | 1.62 | 1.34 | 1.25 | 1.39 | 1.46 | 1.39 | 1.34 | 1.44 | 1.44 | 1.20 |
| 95%CI LL | 1.15 | 1.02 | 0.96 | 0.96 | 0.73 | 0.78 | 1.03 | 0.86 | 0.72 | 0.76 | 0.81 | 0.71 |
| 95%CI UL | 1.89 | 1.70 | 1.69 | 1.56 | 1.30 | 1.41 | 1.70 | 1.50 | 1.34 | 1.43 | 1.47 | 1.27 |

Supplementary Table 3: Descriptive statistics and 95% CIs (LL = lower limit, UL = upper limit) for individual ACQ items (part 2)

|  | Week 1 | Week 2 | Week 3 | Week 4 | Week 5 | Week 6 | Week 7 | Week 8 | Week 9 | Week 10 | Week 11 | Week 12 |
| --- | --- | --- | --- | --- | --- | --- | --- | --- | --- | --- | --- | --- |
| **Night** |  |  |  |  |  |  |  |  |  |  |  |  |
| N | 75 | 76 | 75 | 76 | 75 | 75 | 74 | 73 | 72 | 71 | 72 | 71 |
| Mean | 1.01 | 0.86 | 0.88 | 0.87 | 0.81 | 0.69 | 0.85 | 0.82 | 0.81 | 0.72 | 0.79 | 0.72 |
| Std. Error of Mean | 0.15 | 0.15 | 0.16 | 0.14 | 0.13 | 0.13 | 0.14 | 0.14 | 0.14 | 0.14 | 0.14 | 0.13 |
| Std. Deviation | 1.32 | 1.28 | 1.35 | 1.20 | 1.11 | 1.15 | 1.22 | 1.17 | 1.19 | 1.15 | 1.15 | 1.14 |
| 95%CI LL | 0.71 | 0.57 | 0.58 | 0.60 | 0.56 | 0.43 | 0.57 | 0.55 | 0.53 | 0.45 | 0.53 | 0.45 |
| 95%CI UL | 1.31 | 1.14 | 1.18 | 1.14 | 1.06 | 0.95 | 1.13 | 1.09 | 1.08 | 0.99 | 1.06 | 0.98 |
| **Morning** |  |  |  |  |  |  |  |  |  |  |  |  |
| N | 75 | 76 | 76 | 75 | 75 | 75 | 73 | 73 | 72 | 71 | 72 | 71 |
| Mean | 1.28 | 1.18 | 1.08 | 1.15 | 0.97 | 0.97 | 1.30 | 1.08 | 1.00 | 0.96 | 0.93 | 0.87 |
| Std. Error of Mean | 0.14 | 0.14 | 0.15 | 0.15 | 0.13 | 0.14 | 0.16 | 0.16 | 0.14 | 0.14 | 0.14 | 0.13 |
| Std. Deviation | 1.23 | 1.24 | 1.29 | 1.28 | 1.15 | 1.25 | 1.40 | 1.33 | 1.19 | 1.19 | 1.17 | 1.05 |
| 95%CI LL | 1 | 0.91 | 0.79 | 0.86 | 0.71 | 0.69 | 0.98 | 0.78 | 0.73 | 0.68 | 0.66 | 0.63 |
| 95%CI UL | 1.56 | 1.46 | 1.37 | 1.44 | 1.23 | 1.26 | 1.62 | 1.39 | 1.27 | 1.23 | 1.20 | 1.12 |
| **Restriction** |  |  |  |  |  |  |  |  |  |  |  |  |
| N | 75 | 76 | 76 | 76 | 75 | 75 | 74 | 73 | 72 | 71 | 72 | 71 |
| Mean | 1.36 | 1.11 | 1.09 | 1.03 | 1.03 | 1.04 | 1.24 | 1.08 | 0.94 | 0.92 | 0.90 | 0.90 |
| Std. Error of Mean | 0.15 | 0.14 | 0.16 | 0.16 | 0.15 | 0.16 | 0.18 | 0.16 | 0.15 | 0.16 | 0.14 | 0.14 |
| Std. Deviation | 1.33 | 1.22 | 1.38 | 1.36 | 1.26 | 1.42 | 1.52 | 1.38 | 1.23 | 1.36 | 1.18 | 1.21 |
| 95%CI LL | 1.06 | 0.83 | 0.78 | 0.72 | 0.74 | 0.72 | 0.90 | 0.77 | 0.66 | 0.60 | 0.63 | 0.62 |
| 95%CI UL | 1.66 | 1.38 | 1.40 | 1.33 | 1.31 | 1.36 | 1.59 | 1.40 | 1.23 | 1.23 | 1.17 | 1.18 |
| **Short breath** |  |  |  |  |  |  |  |  |  |  |  |  |
| N | 75 | 75 | 74 | 76 | 74 | 75 | 74 | 73 | 72 | 71 | 72 | 71 |
| Mean | 1.64 | 1.45 | 1.24 | 1.22 | 1.16 | 1.35 | 1.49 | 1.19 | 1.14 | 1.10 | 1.10 | 1.11 |
| Std. Error of Mean | 0.15 | 0.14 | 0.14 | 0.14 | 0.14 | 0.16 | 0.18 | 0.16 | 0.13 | 0.15 | 0.13 | 0.14 |
| Std. Deviation | 1.34 | 1.21 | 1.24 | 1.26 | 1.23 | 1.41 | 1.51 | 1.33 | 1.14 | 1.27 | 1.13 | 1.18 |
| 95%CI LL | 1.34 | 1.18 | 0.96 | 0.94 | 0.88 | 1.03 | 1.14 | 0.89 | 0.88 | 0.80 | 0.84 | 0.84 |
| 95%CI UL | 1.94 | 1.73 | 1.52 | 1.51 | 1.44 | 1.67 | 1.83 | 1.50 | 1.40 | 1.39 | 1.36 | 1.39 |
